# Standby extracorporeal membrane oxygenation: a better strategy for high-risk percutaneous coronary intervention

**DOI:** 10.3389/fmed.2024.1404479

**Published:** 2024-06-26

**Authors:** Chuang Liu, Xingxing Li, Jun Li, Deliang Shen, Qianqian Sun, Junjie Zhao, Hui Zhao, Guowei Fu

**Affiliations:** ^1^Department of Extracorporeal Life Support Center, The First Affiliated Hospital of Zhengzhou University, Zhengzhou, Henan, China; ^2^Department of Cardiovascular Medicine, The First Affiliated Hospital of Zhengzhou University, Zhengzhou, Henan, China

**Keywords:** percutaneous coronary intervention, cardiac arrest, extracorporeal cardiopulmonary resuscitation, standby extracorporeal membrane oxygenation, low-flow time

## Abstract

**Background:**

The incidence of cardiac arrest (CA) during percutaneous coronary intervention (PCI) is relatively rare. However, when it does occur, the mortality rate is extremely high. Extracorporeal cardiopulmonary resuscitation (ECPR) has shown promising survival rates for in-hospital cardiac arrests (IHCA), with low-flow time being an independent prognostic factor for CA. However, there is no definitive answer on how to reduce low-flow time.

**Methods:**

This retrospective study, conducted at a single center, included 39 patients who underwent ECPR during PCI between January 2016 and December 2022. The patients were divided into two cohorts based on whether standby extracorporeal membrane oxygenation (ECMO) was utilized during PCI: standby ECPR (SBE) (*n* = 13) and extemporaneous ECPR (EE) (*n* = 26). We compared the 30-day mortality rates between these two cohorts and investigated factors associated with survival.

**Results:**

Compared to the EE cohort, the SBE cohort showed significantly lower low-flow time (*P* < 0.01), ECMO operation time (*P* < 0.01), and a lower incidence of acute kidney injury (AKI) (*P* = 0.017), as well as peak lactate (*P* < 0.01). Stand-by ECMO was associated with improved 30-day survival (*p* = 0.036), while prolonged low-flow time (*p* = 0.004) and a higher SYNTAX II score (*p* = 0.062) predicted death at 30 days.

**Conclusions:**

Standby ECMO can provide significant benefits for patients who undergo ECPR for CA during PCI. It is a viable option for high-risk PCI cases and may enhance the overall prognosis. The low-flow time remains a critical determinant of survival.

## 1 Introduction

The incidence of cardiac arrest (CA) during percutaneous coronary intervention (PCI) is relatively low, estimated at around 1.5% (1, 2). However, despite a higher likelihood of successful resuscitation compared to other in-hospital cardiac arrest (IHCA) scenarios (3), it is still associated with the highest mortality rates (4, 5). Advancements in PCI techniques and mechanical circulatory support devices have enabled interventional cardiologists to attempt revascularization of more intricate coronary anatomy in patients who are often ineligible for surgical intervention (6). However, this increased complexity also brings about a higher risk of PCI-related CA. The efficacy of extracorporeal membrane oxygenation (ECMO) for patients undergoing PCI has been demonstrated in numerous studies (7–10). Nonetheless, incorporating routine ECMO usage to support the growing number of high-risk PCIs would not only result in heightened patient trauma and complications but also place a financial burden and strain on medical resources.

Whether it pertains to cardiogenic shock, cardiac arrest, or perioperative support for PCI, veno-arterial extracorporeal membrane oxygenation ECMO (VA ECMO) has demonstrated significant effectiveness (7–9). Shaukat et al. (10) have demonstrated that VA ECMO can provide adequate hemodynamic support for high-risk PCI patients. Research has demonstrated that the routine use of VA ECMO in high-risk patients with percutaneous transluminal coronary angioplasties (PTCA) is a positive prognostic factor for patient survival (11). Nonetheless, implementing routine ECMO usage to support the growing number of high-risk PCI would not only lead to increased patient trauma and complications but also impose a financial burden and strain on medical resources. Furthermore, the current landscape lacks universally acknowledged criteria for the routine employment of ECMO. In our previous study, we established that adopting a standby ECMO strategy during high-risk PCI effectively serves as an emergency rescue measure and yields satisfactory outcomes in the event of cardiac arrest (3). Recognizing the critical importance of salvage time during such emergencies, we endeavored to further reduce the rescue time with the objective of improving survival rates.

## 2 Materials and methods

### 2.1 Patient selection

This is a single-center retrospective study that includes all cases of Extracorporeal Cardiopulmonary Resuscitation (ECPR) during PCI procedures from January 2016 to December 2022. The study included a total of 39 participants, who were divided into two cohorts based on whether standby ECMO was utilized during PCI: standby ECPR (SBE) (*n* = 13) and extemporaneous ECPR (EE) (*n* = 26).

Data related to clinical, angiographic, procedural, and outcome variables were collected from the hospital's medical records. In the high-risk PCI cohort, the HeartTeam makes the decision to utilize standby extracorporeal membrane oxygenation. Patients meeting the following criteria were eligible: coronary artery disease of the left main, a single remaining conduit, or severe multivessel disease, taking the SYNTAX score into account; with a severely impaired left ventricular ejection fraction (LVEF), defined as LVEF ≤ 35% or decompensated heart failure, defined as the presence of heart failure with clinical symptoms necessitating treatment; rejected for CABG as a primary treatment option (12). The study protocols were approved by the Ethics Committee of the First Affiliated Hospital of Zhengzhou University (2023-KY-0638). Due to the retrospective and observational nature of the study, written informed consent was waived.

### 2.2 The procedure for VA-ECMO connection and management

The ECMO circuit consisted of a centrifugal pump (Rotaflow; MAQUET Cardiovascular, Hirlingen, Germany), a polymethylpentene oxygenator (Quadrox PLS; MAQUET Cardiovascular), and Bio-Medicus^®^ Femoral Venous and Bio-Medicus^®^ Femoral Arterial cannulas (Medtronic Inc, Minneapolis, MN) for insertion into the patient's vascular system.

Bio-Medicus^®^ Femoral Arterial cannulas with diameters ranging from 15 to 19 French and Bio-Medicus^®^ Femoral Venous cannulas ranging from 19 to 25 French were selected based on the patients' biometric data. The distal end of the arterial cannula was positioned in the common iliac artery, while the distal end of the venous cannula was situated in the right atrium. The hypocoagulant state was achieved by maintaining the activated coagulation time between 180 and 220 s through continuous intravenous infusion of unfractionated heparin. Bolus dosing of unfractionated heparin was excluded due to its prior administration during PCI.

In the SBE cohort, the ECMO circuit was prepared for connection and priming with Multiple Electrolytes Injection (Shanghai Baxter Medical Supplies Co., Ltd., Lot No. S2104020, Specification 500 ml) prior to PCI in the cardiac catheterization laboratory (CCL). Cannula sizes were predetermined based on the desired flow rate and femoral artery diameter, assessed through ultrasound. Subsequently, two 5 French catheters were inserted into the femoral artery and femoral vein, respectively, using an ultrasound-assisted Seldinger technique after obtaining consent for ECMO. In instances of CA immediate initiation of cardiopulmonary resuscitation (CPR) would be followed by the insertion of the ECMO arterial cannula into the femoral artery through the pre-inserted 5 French catheter after the dilatation procedure. The venous cannula was inserted in a similar manner. After meticulously removing air from the system, the primed ECMO circuit will be interconnected, promptly initiating ECMO support, and proceeding to PCI, without the need to re-sign the informed consent form.

In cases of CA occurring in the EE cohort during PCI, immediate CPR and a distress call were made to the ECMO team, who promptly responded to the CCL. The patient's guardian was then required to provide consent for ECMO support after the ECMO team arrived. The ECMO circuit was connected and primed while simultaneously puncturing the femoral artery and femoral vein for the appropriate tube insertion. After meticulously removing air from the system, the ECMO circuit was interconnected to promptly initiate ECMO support, facilitating the continuation of PCI procedures.

The initial flow rate of ECMO was 2.0–2.2 L/(min·m2), with subsequent adjustments made based on blood pressure to maintain a mean arterial pressure of ≥65 mmHg. Prompt placement of a distal perfusion catheter (six French) was essential to ensure adequate blood supply to the ipsilateral limb in case of ischemic symptoms resulting from femoral artery cannulation. Disconnection from ECMO was considered when the patient was hemodynamically stable at an ECMO flow rate of < 1 L/(min·m2).

### 2.3 Statistical analysis

Continuous variables were presented as mean ± standard deviation and analyzed using the unpaired *t*-test. If continuous variables showed a skewed distribution, they were presented as quartiles and compared using the Mann-Whitney *U*-test. Categorical variables were expressed as percentages and compared using Pearson's χ^2^ test or Fisher's exact test. Adjusted logistic regression models were used to assess clinical outcomes, accounting for relevant covariates. Variables that showed significance (*p* < 0.1) in the univariate analysis were included in the multivariate analysis. Statistical significance was defined as a *p*-value < 0.05 (all tests were two-sided). Statistical analyses were conducted using SPSS for Windows version 21.0 (SPSS Inc., Chicago, IL, USA).

## 3 Results

### 3.1 Baseline characteristics

**Figure 1:**
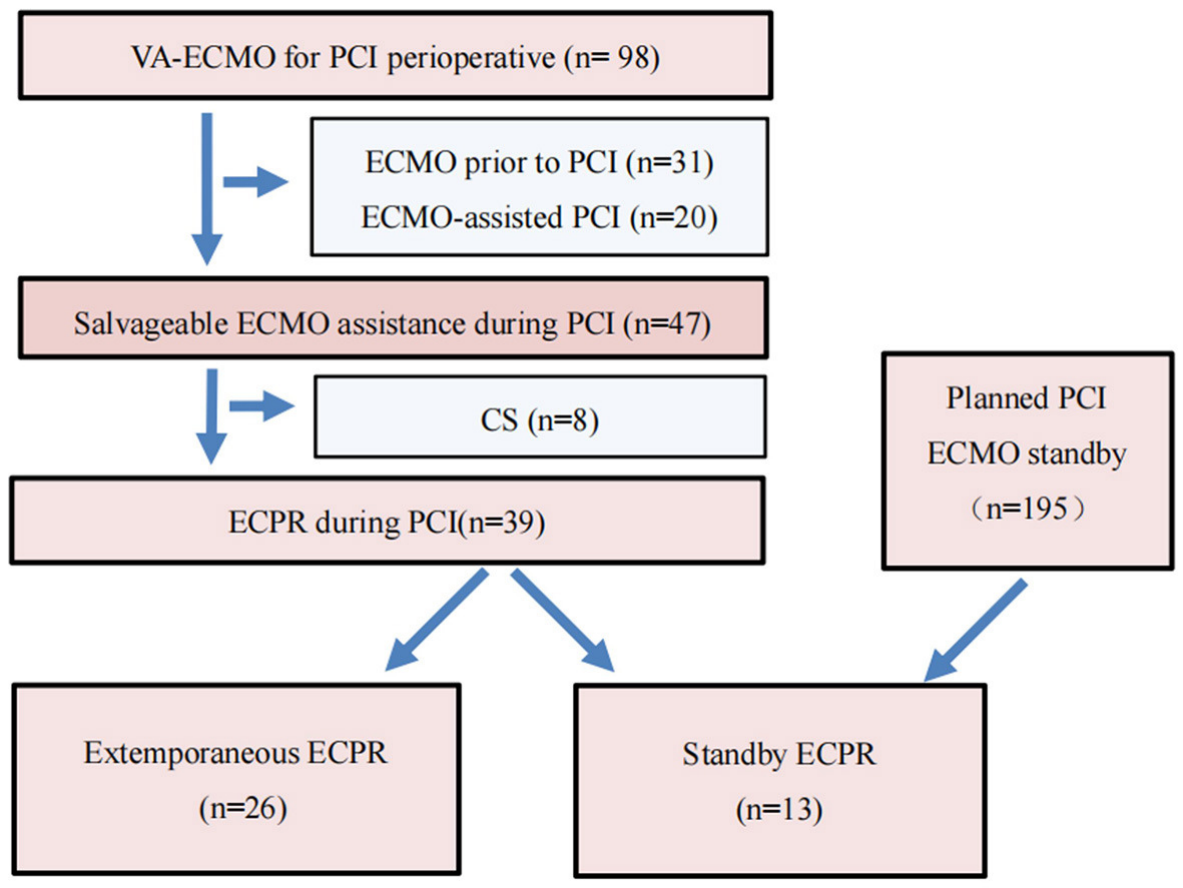
Flow diagram of VA-ECMO for PCI perioperative. VA-ECMO, veno-arterial extracorporeal membrane oxygenation; PCI, percutaneous coronary intervention; ECPR, extracorporeal cardiopulmonary resuscitation; SBE, “Stand-by” ECPR; EE, extemporaneous ECPR.

A total of 98 patients underwent VA-ECMO for perioperative PCI. Among them, 31 patients received emergency VA-ECMO support prior to PCI due to circulatory failure, 20 patients underwent ECMO-assisted PCI, and 47 patients received salvageable ECMO assistance during PCI. During the PCI procedure, eight patients experienced cardiogenic shock, while extracorporeal cardiopulmonary resuscitation (ECPR) was performed on 39 patients. These 39 patients were further divided into two groups: 13 patients underwent standby ECMO (SBE) (a total of 195 patients underwent standby ECMO during this period) with a mean age of 56.62 ± 15.04 years, while the remaining 26 patients received extemporaneous ECPR (EE) with a mean age of 59.39 ± 11.14 years during PCI (Figure 1). The baseline characteristics of the cohorts are presented in Table 1.

**Table 1:** Baseline characteristics of patients in the SBE and EE groups.

**Variables**	**SBE (*n* = 13)**	**EE (*n* = 26)**	***P*-value**
**Baseline characteristics**			
Age (years)	56.62 ± 15.04	59.39 ± 11.14	0.519
Male (sex), *n* (%)	11 (84.6%)	19 (73.1%)	0.687
BMI (kg/m^2^)	23.98 ± 3.5	25.36 ± 2.32	0.149
Smoking, *n* (%)	9 (69.2%)	9 (34.6%)	0.105
Hypertension, *n* (%)	6 (46.2%)	18 (69.2%)	0.163
Diabetes mellitus, *n* (%)	5 (38.5%)	13 (50%)	0.496
Hyperlipidemia, *n* (%)	0 (0%)	4 (15.4%)	0.351
Previous CHD, *n* (%)	9 (69.2%)	11 (42.3%)	0.113
Previous cardiac surgery and/or cardiac intervention, *n* (%)	3 (23.1%)	7 (26.9%)	1.000
Previous CRF, *n* (%)	1 (7.7%)	3 (11.5%)	1.000
Previous stroke, *n* (%)	3 (23.1%)	9 (34.6%)	0.713
Peripheral arterial disease, *n* (%)	1 (7.7%)	1 (3.8%)	1.000

SBE, “Stand-by” ECPR; EE, extemporaneous ECPR; BMI, body mass index; CHD, coronary heart disease; CRF, chronic renal failure.

### 3.2 Procedural characteristics of patients in the SBE and EE groups

Notably, there was a significant difference between the cohorts in terms of lower preoperative left ventricular ejection fraction (LVEF) at 32.85% in the SBE cohort compared to 41.73% in the EE cohort (*p* = 0.015). Additionally, the SBE cohort exhibited significantly lower low-flow time and ECMO operation time compared to the EE cohort (5.54 vs. 37.65 min, *p* = 0.000; 7.15 vs. 20.50 min, *p* = 0.000). The SBE cohort had significantly lower peak lactate values (4.59 vs. 13.38 mmol/L, *p* = 0.000) and a lower incidence of acute kidney injury acute kidney injury (AKI) AKI diagnoses (0.0 vs. 42.3%, *p* = 0.017) (Table 2).

**Table 2:** Procedural characteristics and in-hospital outcomes of patients in the SBE and EE groups.

**Variables**	**SBE (*n* = 13)**	**EE (*n* = 26)**	***P*-value**
**Procedure characteristics**			
**Infarct-related coronary artery**			
Left anterior descending, *n* (%)	12 (92.3%)	24 (92.3%)	1.000
Left circumflex, *n* (%)	9 (69.2%)	18 (69.2%)	1.000
Right, *n* (%)	10 (76.9%)	19 (73.1%)	1.000
Left main, *n* (%)	4 (30.8%)	10 (38.5%)	0.906
SYNTAX score	44.65 ± 15.47	42.35 ± 16.07	0.671
SYNTAX II score	49.29 ± 16.71	46.05 ± 12.91	0.507
Euro score	44.87 ± 28.49	32.7 ± 25.88	0.189
Euro II score	19.39 ± 9.89	15.62 ± 19.59	0.430
EF (%)	32.85 ± 8.53	41.73 ± 12.85	0.015
EF < 35, *n* (%)	7 (53.8%)	9 (34.6%)	0.250
IABP, *n* (%)	8 (61.5%)	12 (46.2%)	0.365
**Heart rhythm in cardiac arrest**			
Shockable rhythm, *n* (%)	13 (100%)	20 (76.9%)	0.158
Ventricular standstill, *n* (%)	0 (0%)	3 (11.5%)	0.524
PEA, *n* (%)	0 (0%)	3 (11.5%)	0.524
Low-flow time	5.54 ± 1.56	37.65 ± 10.32	0.000
ECMO operation time	7.15 ± 2.7	20.5 ± 5.77	0.000
DPC, *n* (%)	5 (38.5%)	10 (38.5%)	1.000
Peak Lactate (mmol/L)	4.59 ± 1.5	13.38 ± 3.87	0.000
**In-hospital outcomes**			
Successful ECMO weaning, *n* (%)	11 (84.6%)	15 (57.7%)	0.186
30-day survival, *n* (%)	11 (84.6%)	13 (50%)	0.036
Duration of ECMO (h)	63.19 ± 41.26	60.37 ± 51.8	0.865
Limb complications, *n* (%)	2 (15.4%)	8 (30.8%)	0.517
Cannulation site Bleeding, *n* (%)	0 (0%)	4 (15.4%)	0.351
Leg ischemia, *n* (%)	1 (7.7%)	2 (7.7%)	1.000
Pseudoaneurysm, *n* (%)	1 (7.7%)	2 (7.7%)	1.000
Airway bleeding, *n* (%)	0 (0%)	2 (7.7%)	0.544
Gastrointestinal bleeding, *n* (%)	0 (0%)	3 (11.5%)	0.524
AKI, *n* (%)	0 (0%)	11 (42.3%)	0.017
CRRT, *n* (%)	4 (30.8%)	9 (34.6%)	1.000
CPC (1–2), *n* (%)	11 (100%)	11 (84.6%)	0.482

SBE, “Stand-by” ECPR; EE, extemporaneous ECPR; EF, ejection fraction; IABP, intra-aortic balloon pump; PEA, pulseless electrical activity; ECMO, extracorporeal membrane oxygenation; DPC, distal perfusion catheter; AKI, acute kidney injury; CRRT, continuous renal replacement therapy; CPC, cerebral performance category.

### 3.3 Clinical outcomes

**Figure 2:**
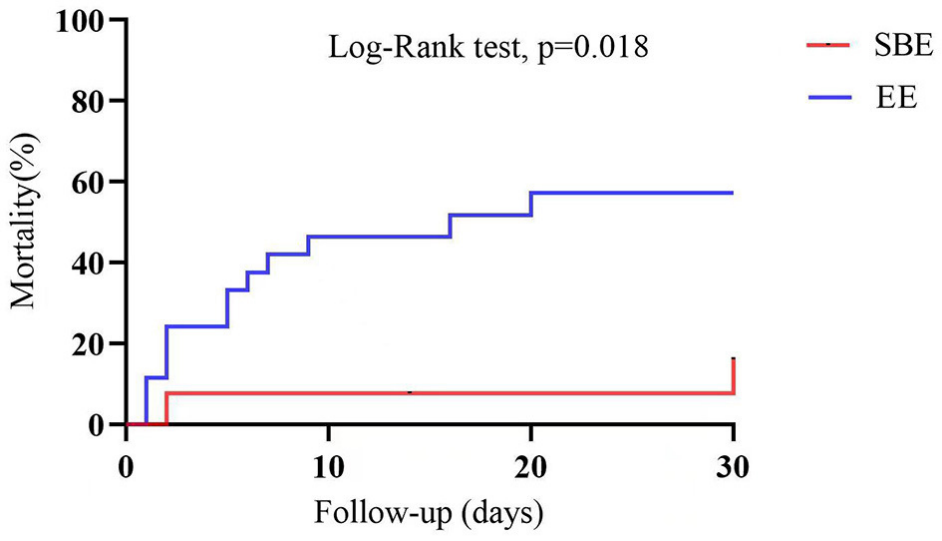
Analysis of 30-day survival curves for patients in the SBE group and the EE group. SBE, “Stand-by” ECPR; EE, extemporaneous ECPR.

The cohorts demonstrated similar ECMO duration (SBE: 63.19 h vs. EE: 60.37 h, *p* = 0.865, Table 2). However, there were statistically significant differences between the groups in terms of successful weaning rate from ECMO and 30-day survival (84.6 vs. 57.7%, *p* = 0.186; 84.6 vs. 50.0%, *p* = 0.036, Table 2), and 30-day survival was similar on Kaplan-Meier survival analysis (log-rank *p* = 0.018, Figure 2).

In the SBE cohort, two patients failed to be weaned from ECMO due to multiple organ dysfunction syndrome (MODS) and intractable cardiogenic shock. Among the patients successfully weaned from ECMO in this group, all survived until the 30-day follow-up with a favorable neurological status. In the EE cohort, 11 cases failed to wean from ECMO due to various reasons, including MODS (4), brain death (3), gastrointestinal tract bleeding (1), cerebral ischemia (1), and intractable cardiogenic shock (2). Out of the 15 patients who successfully withdrew from ECMO, two did not survive at the 30-day follow-up due to MODS and cardiac arrest. Among the remaining 13 survivors, eleven (84.6%) exhibited a favorable neurological status.

Overall, the survivors had lower SYNTAX II scores (43.22 vs. 53.39, *p* = 0.027, Table 3) and peak lactate levels (8.98 vs. 12.81 mmol/L, *p* = 0.026, Table 3), a higher utilization of standby ECMO support (45.8 vs. 13.3%, *p* = 0.036, Table 3), shorter low-flow time (20.88 vs. 36.67 min, *p* = 0.005, Table 3), and ECMO operation time (14.04 vs. 19.27 min, *p* = 0.047, Table 3), a greater success rate in weaning from ECMO (100 vs. 13.3%, *p* = 0.000, Table 3), a reduced incidence of limb complications (12.5 vs. 46.7%, *p* = 0.045, Table 3), as well as a lower prevalence of continuous renal replacement therapy (CRRT) requirements (16.7 vs. 60.0%, *p* = 0.005, Table 3). Limb complications were more frequent in the group of patients who did not survive compared to those who survived (12.5 vs. 46.7%, *p* = 0.045, Table 3).

**Table 3:** Comparison of procedural characteristics and in-hospital outcomes between survivors and non-survivors.

**Variables**	**Survival (*n* = 24)**	**Death (*n* = 15)**	***P*-value**
**Procedure characteristics**			
**Infarct-related coronary artery**			
Left anterior descending	22 (91.7%)	14 (93.3%)	1.000
Left circumflex	17 (70.8%)	10 (66.7%)	1.000
Right	19 (79.2%)	10 (66.7%)	0.622
Left main	9 (37.5%)	5 (33.3%)	0.792
SYNTAX score	41.1 ± 16	46.33 ± 15.19	0.318
SYNTAX II score	43.22 ± 13.03	53.39 ± 14.01	0.027
Euro score	31.07 ± 24.13	45.87 ± 29.7	0.097
Euro II score	16.78 ± 17.34	17.03 ± 16.84	0.965
EF (%)	37.92 ± 12.24	40.13 ± 12.53	0.589
EF < 35%	10 (41.7%)	6 (40%)	0.918
IABP	12 (50%)	8 (53.3%)	0.839
**Heart rhythm in cardiac arrest**			
Shockable rhythm	22 (91.7%)	11 (73.3%)	0.277
Ventricular standstill	1 (4.2%)	2 (13.3%)	0.669
PEA	1 (4.2%)	2 (13.3%)	0.669
“Stand-by” ECMO	11 (45.8%)	2 (13.3%)	0.036
Low-flow time	20.88 ± 15.09	36.67 ± 17.1	0.005
ECMO operation time	14.04 ± 8.17	19.27 ± 6.94	0.047
DPC	8 (33.3%)	7 (46.7%)	0.405
Peak Lactate (mmol/L)	8.98 ± 5.09	12.81 ± 4.92	0.026
**In-hospital outcomes**			
Successful ECMO weaning	24 (100%)	2 (13.3%)	0.000
Duration of ECMO (h)	60.44 ± 36.57	62.69 ± 63.69	0.889
Limb complications, *n* (%)	3 (12.5%)	7 (46.7%)	0.045
Cannulation site Bleeding, *n* (%)	2 (8.3%)	2 (13.3%)	1.000
Leg ischemia, *n* (%)	0	3(17.6%)	0.108
Pseudoaneurysm, *n* (%)	1 (4.2%)	2 (13.3%)	0.669
Airway bleeding, *n* (%)	0 (0%)	2 (13.3%)	0.142
Gastrointestinal bleeding, *n* (%)	2 (8.3%)	1 (6.7%)	1.000
AKI	4 (16.7%)	7 (46.7%)	0.097
CRRT	4 (16.7%)	9 (60%)	0.005

SBE, “Stand-by” ECPR; EE, extemporaneous ECPR; EF, ejection fraction; IABP, intra-aortic balloon pump; PEA, pulseless electrical activity; ECMO, extracorporeal membrane oxygenation; DPC, distal perfusion catheter; AKI, acute kidney injury; CRRT, continuous renal replacement therapy; CPC, cerebral performance category.

### 3.4 The relationship between low-flow time and 30-day outcomes

**Figure 3:**
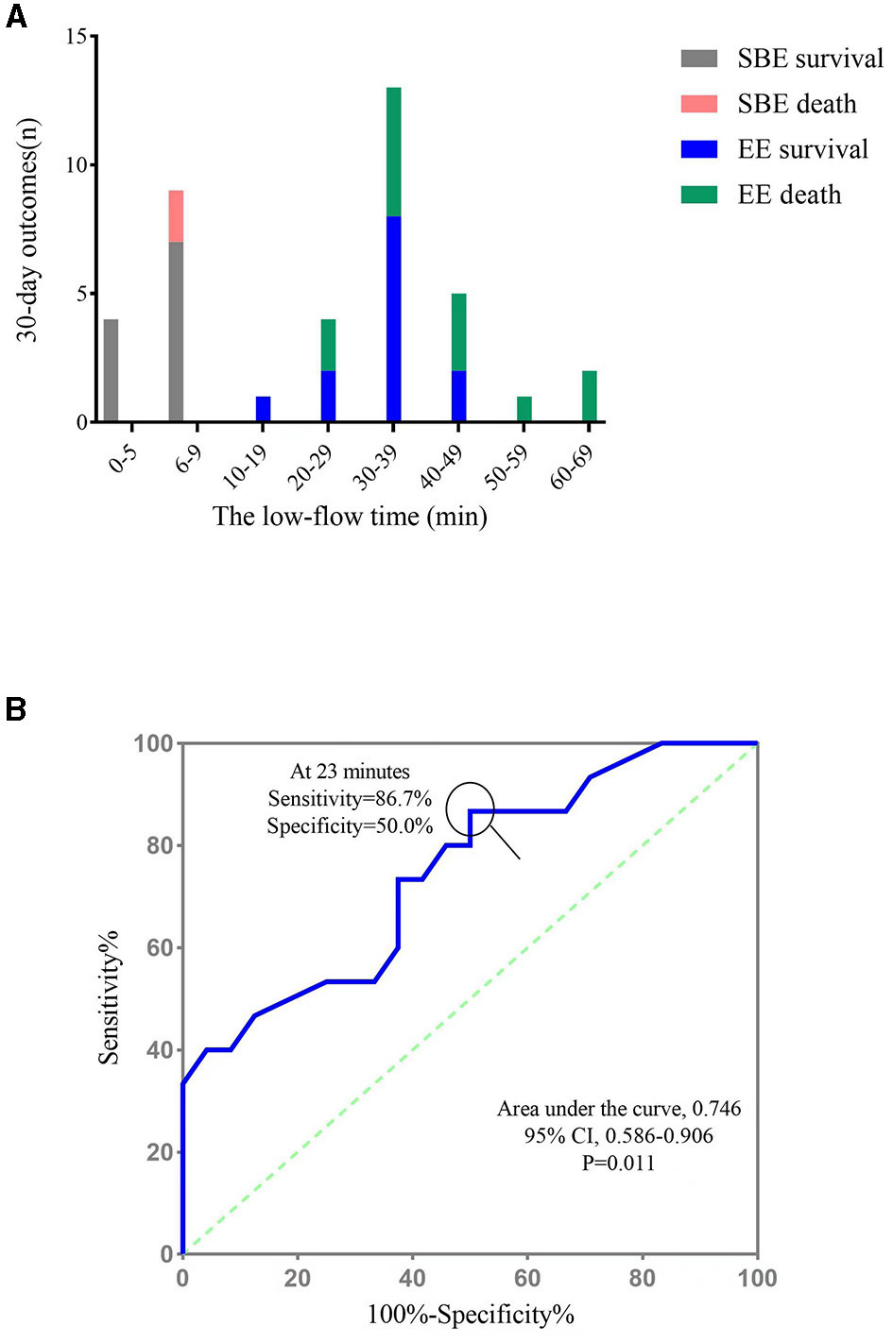
The relationship between low low-flow time and 30-day outcome. **(A)** The number of survivors and non-survivors in the EE and SBE groups at different low-flow time intervals; **(B)** ROC curve for the low-flow time for the prediction of 30-day mortality. SBE, “Stand-by” ECPR; EE, extemporaneous ECPR.

Figure 3A shows the number of survivors and non-survivors in the EE and SBE groups at different low-flow time intervals. In the SBE group, all patients had a low-flow time of < 10 min. Of these, all four patients with a low-flow time of < 5 min survived, giving a survival rate of 100%, whereas the survival rate for low-flow time of 6–9 min was 77.8% (7/9). However, patients in the EE group experienced low-flow times ranging from 10 to 69 min. Notably, none of the patients with a low flow time of more than 50 min survived to discharge.

ROC curve analysis was performed to define an optimal cut-off CPR time value to predict in-hospital mortality in patients undergoing ECPR during PCI. The area under the curve was 0.746 [95% confidence interval (CI), 0.586–0.906; *p* = 0.011], and the highest accuracy in distinguishing mortality outcomes was achieved at a cut-off of 23 min (86.7% sensitivity and 50.0% specificity, Figure 3B).

### 3.5 Predictors of 30-day mortality

To identify the independent predictors of 30-day mortality, we performed Cox's proportional hazards regression analysis, as shown in Table 4. In the univariate regression analysis, low-flow time showed a significant association with improved 30-day survival (HR 1.05; 95% CI 1.02–1.08; *p* = 0.004). On the other hand, prolonged ECMO operation time (HR 1.11; 95% CI 1.03–1.20; *p* = 0.007), standby ECMO (HR 0.20; 95% CI 0.05–0.90; *p* = 0.036), higher SYNTAX II score (HR 1.03; 95% CI 1.00–1.06; *p* = 0.062), elevated peak lactate levels (HR 1.10; 95% CI 1.01–1.20; *p* = 0.037), and a high incidence of AKI (HR 0.25; 95% CI 0.09–0.71; *p* = 0.009), as well as the use of continuous renal replacement therapy (CRRT) (HR 0.25; 95% CI 0.09–0.70; *p* = 0.009), were identified as significant predictors of mortality at 30 days. In the multivariate regression analysis, a higher SYNTAX II score (HR 1.04; 95% CI 1.01–1.08; *p* = 0.023) and increased low-flow time (HR 1.05; 95% CI 1.02–1.08; *p* = 0.001) were found to be significantly associated with an increased risk of 30-day mortality.

**Table 4:** Predictors of death at 30-days.

**Variables**	**HR (95% CI)**	***P*-value**
**Univariate analysis**		
Standby ECMO	0.16 (0.04–0.68)	0.014
Age (years)	1.01 (0.96–1.06)	0.736
SYNTAX II score	1.06 (1.01–1.11)	0.029
ECMO operation time	1.16 (1.04–1.30)	0.008
AKI	13.64 (1.46–127.15)	0.022
CRRT	4.73 (1.21–18.47)	0.026
Low-flow time	1.07 (1.02–1.12)	0.004
Peak lactate (mmol/L)	1.15 (1.01–1.31)	0.029
Limb complications	10.67 (1.89–60.08)	0.007
**Multivariate analysis**		
SYNTAX II score	1.10 (1.03–1.18)	0.006
Low-flow time	1.11 (1.03–1.19)	0.004

CI, confidence interval; ECMO, extracorporeal membrane oxygenation; AKI, acute kidney injury; CRRT, continuous renal replacement therapy; HR, hazard ratio.

## 4 Discussion

This study aimed to evaluate the baseline characteristics, clinical outcomes, and complications in 39 patients who underwent extracorporeal cardiopulmonary resuscitation (ECPR) during PCI. The main findings of this study are as follows: (1) Despite the successful implementation of ECPR and complete revascularization in patients experiencing cardiac arrest during PCI, the 30-day mortality rate remained high. (2) Standby ECMO was found to significantly reduce low-flow time and ECMO operating time compared to extemporaneous ECMO. (3) Patients who received standby ECMO during PCI showed lower 30-day mortality rates and improved neurological outcomes.

PCI in high-risk patients is associated with several complications, including no coronary artery reflow, coronary artery dissection, pericardial tamponade, hemodynamic instability, and CA (13). Currently, there is no standardized protocol for high-risk PCI, and determining the need for hemodynamic support can be challenging. The use of mechanical circulatory support (MCS) devices during high-risk PCIs is believed to reduce the potential risks associated with major adverse events both during and post-revascularization procedures. Among the most prevalent MCS devices employed are the intra-aortic balloon pump (IABP), veno-arterial extracorporeal membrane oxygenation (V-A ECMO), and the Impella. However, there are inconsistencies in findings across small-scale studies, registries, and randomized clinical trials regarding the benefits of MCS in high-risk PCIs. Furthermore, the available data on the utilization of IABP and ECMO is either insufficient or outdated, thus failing to accurately represent current interventional practices (14). Considering the possibility of hemodynamic instability or cardiac arrest during high-risk PCI, the use of ECMO can provide robust circulatory support and significantly improve patient prognosis (6, 7, 15, 16). However, VA-ECMO may also increase the risk of complications, such as elevated cardiac afterload, bleeding, lower limb arterial ischemia, hemolysis, AKI, and a higher susceptibility to infections (7, 17–21). As there is currently limited clinical data on using VA-ECMO as mechanical circulatory support MCS during high-risk PCI, guidelines do not include specific recommendations. The potential increase in procedure time, patient discomfort, and expenses associated with prophylactic ECMO use would require clear evidence of its benefits before it can be widely adopted.

Studies have shown that extracorporeal cardiopulmonary resuscitation (ECPR) treatment for in-hospital cardiac arrests (IHCA) has yielded promising survival rates ranging from 20 to 45% (22, 23). Various studies, including those conducted in emergency departments, pre-hospital settings, and CCL, have demonstrated the efficacy of ECPR (24–28). The timing of interventions is a critical factor influencing survival rates. Each additional 10 min of CPR beyond the initial 30 min leads to a 25% decrease in survival for ECPR (29). A study by Chen et al. found that the probability of surviving until discharge was 50, 30, and 10% for low-flow times of 30, 60, and 90 min, respectively (30). In our present study, the SBE cohort exhibited a lower low-flow time (5.54 ± 1.56), which was significantly reduced through the use of standby ECMO. Furthermore, this strategy demonstrated advantages in terms of 30-day survival and favorable neurological outcomes. These findings provide compelling evidence to reconsider the standby of MCS in high-risk PCI cases.

In our study, the highest level of accuracy in distinguishing mortality outcomes was attained at an E-CPR time of 23 min. According to the guidelines established by the Extracorporeal Life Support Organization (ELSO), it is recommended to consider initiating cannulation for extracorporeal cardiopulmonary resuscitationECPR after 10–20 min of unsuccessful resuscitation efforts (31). Given the cannulation time required for ECMO, the total duration of low-flow time will be significantly extended, which may result in an increased mortality rate. Moreover, in cases of cardiac arrest (CAs) occurring during PCI procedures, although prompt cardiopulmonary resuscitation (CPR) may facilitate the restoration of spontaneous circulation, there remains a potential need for subsequent PCI, which is associated with significant risks. Therefore, it is advised that in such circumstances, prompt initiation of ECMO support should be prioritized without delay, rather than waiting for a period of 10–20 min.

Ultrasound guidance combined with fluoroscopic verification of wire positioning has been reported to achieve high rates of successful cannulation with minimal vascular complications (32, 33). However, even at high-volume centers, rates of vascular complications can still be significant. Providers should be prepared for these potentially serious adverse events, especially in the context of extracorporeal cardiopulmonary resuscitation (ECPR) due to its emergent nature (34). A study has demonstrated that the prevalence of limb ischemia among patients undergoing out-of-hospital cardiac arrest is 29.5% (35). In our study, the incidence of limb complications was 25.6%, with rates of 15.4% in the SBE group and 30.8% in the EE group. The most common complication identified was bleeding at the intubation site, which exclusively occurred in patients from the EE group. This finding can be attributed to meticulous preparation and adequate time allocation during standby ECMO intubation, while impromptu ECMO procedures were associated with a higher risk of complications.

The incidence of AKI in patients receiving ECMO treatment varies widely, ranging from 26 to 85%, depending on patient characteristics, the definition of AKI, and clinical settings. Severe AKI requiring renal replacement therapy (RRT) has a pooled estimated incidence of 45% (36). AKI is associated with a high mortality rate in patients receiving VA -ECMO. It is unclear whether AKI's presence is purely prognostic, related to the severe injury caused by cardiac arrestCA, or if it directly contributes to mortality. In our study, all patients underwent coronary angiography, which involved the use of intravenous contrast media, known to potentially contribute to kidney injury. The overall incidence of AKI was 28.2%, with a rate of 0% in the SBE group and 42.3% in the EE group. This difference may be due to the significantly reduced low-flow time achieved through standby ECMO. Additionally, our findings suggest that AKI is a significant prognostic factor in patients undergoing ECPR during PCI. A study on the use of blood lactate levels to predict 30-day mortality in patients receiving VA ECMO treatment for refractory cardiogenic shock (RCS) or refractory cardiac arrest CA (RCA) complicating acute coronary syndrome (ACS) found that non-survivors exhibited significantly elevated blood lactate levels within the first 24 h (37). The peak lactate levels were significantly lower in the SBE cohort compared to the EE cohort in our study, however, this difference did not predict 30-day mortality.

## 5 Limitations

This study was a single-center retrospective study with a small sample size. As with any observational study, selection bias, information bias, and confounding bias are potential limitations. Additionally, the observational nature of the study means that variations in CPR techniques and intensities among cases could have influenced patient outcomes. Not all high-risk PCI procedures were conducted with a standby ECMO system available. The decision to use ECMO as a backup measure was made based on a case-by-case assessment, taking into account clinical evaluation and individual preferences. Consequently, the criteria for assessing the necessity of standby ECMO are not uniform, which may influence the categorization within this research study. We only present data on the 30-day results, excluding information on long-term outcomes. Additionally, a detailed analysis aimed at identifying a cohort of high-risk PCI patients who would benefit from routine circulatory support was not conducted, as the focus of this study is to explore a more effective approach that supersedes the current practice of emergent or extemporaneous ECMO utilization and its routine implementation.

## 6 Conclusions

In this study, standby ECMO provided immediate and reliable hemodynamic support for patients experiencing cardiac arrest during high-risk PCI, as needed. Standby ECMO can significantly reduce the low-flow time and ECMO operation time of ECPR during PCI, thus improving the 30-day survival rate. Therefore, it is a viable option for high-risk PCI cases and may enhance the overall prognosis.

## Data availability statement

The raw data supporting the conclusions of this article will be made available by the authors, without undue reservation.

## Ethics statement

The studies involving humans were approved by the Ethics Committee of the First Affiliated Hospital of Zhengzhou University. The studies were conducted in accordance with the local legislation and institutional requirements. The ethics committee/institutional review board waived the requirement of written informed consent for participation from the participants or the participants' legal guardians/next of kin because due to the retrospective and observational nature of the study, written informed consent was waived.

## Author contributions

CL: Conceptualization, Data curation, Formal analysis, Investigation, Methodology, Project administration, Writing – original draft, Writing – review & editing. XL: Conceptualization, Data curation, Investigation, Methodology, Resources, Software, Supervision, Validation, Visualization, Writing – original draft, Writing – review & editing. JL: Supervision, Validation, Writing – review & editing. DS: Conceptualization, Project administration, Resources, Supervision, Validation, Writing – original draft, Writing – review & editing. QS: Data curation, Investigation, Methodology, Writing – review & editing. JZ: Data curation, Formal analysis, Investigation, Visualization, Writing – review & editing. HZ: Conceptualization, Investigation, Supervision, Validation, Writing – review & editing. GF: Methodology, Resources, Supervision, Validation, Visualization, Writing – review & editing.
